# Age-associated increases in middle cerebral artery pulsatility differ between men and women

**DOI:** 10.1152/ajpheart.00453.2023

**Published:** 2023-09-08

**Authors:** Wesley K. Lefferts, Krista S. Reed, Rachel E. Rosonke, Jacqueline A. Augustine, Kerrie L. Moreau

**Affiliations:** ^1^Department of Kinesiology, Iowa State University, Ames, Iowa, United States; ^2^Kinesiology Department, SUNY Cortland, Cortland, New York, United States; ^3^Division of Geriatric Medicine, University of Colorado Anschutz Medical Campus, Aurora, Colorado, United States

**Keywords:** aging, cerebral pulsatility, large artery stiffness, midlife, sex differences

## Abstract

Mechanisms underlying sex differences in brain aging remain unclear but may relate to changes in cerebral pulsatile blood flow. Sex differences in the stiffening of the large arteries and expansion of pulse pressure with age may accelerate changes in pulsatile (i.e., discontinuous) blood flow in the brain that contribute to brain health. The purpose of this cross-sectional, secondary analysis was to examine sex differences in age-associated changes in large artery (aorta and carotid) stiffness, carotid pulse pressure, and cerebral pulsatility in 206 men and 217 women between 18 and 72 yr of age. Outcomes included aortic stiffness [carotid-femoral pulse wave velocity (cfPWV)] and carotid pulse pressure via tonometry, carotid β-stiffness via ultrasound, and middle cerebral artery (MCA) pulsatility index via transcranial Doppler. Regression analyses revealed a significant age-by-sex interaction, with women exhibiting a slower rate of change compared with men for cfPWV (β = −0.21, *P* = 0.04), and greater rate of change for carotid stiffness (β = 0.27, *P* = 0.02), carotid pulse pressure (β = 0.98, *P* < 0.001), and MCA pulsatility index (β = 0.49, *P* = 0.002) after adjustment for covariates. The significant age-by-sex interaction for MCA pulsatility was abolished after further adjustment for carotid pulse pressure. Women exhibit accelerated increases in cerebral pulsatility during midlife, likely driven by exaggerated increases in carotid stiffness and pulse pressure compared with men. These data suggest that there are disproportionate increases in cerebral pulsatility in women during midlife that could contribute to accelerated brain aging compared with men.

**NEW & NOTEWORTHY** We identify sex-specific associations between increasing age and cerebral pulsatility and its vascular mechanisms. When compared with men, women in our cross-sectional analysis exhibited greater age-associated increases in carotid stiffness, carotid pulse pressure, and cerebral pulsatility particularly during midlife. These data suggest that the rapid expansion of pulse pressure during midlife contributes to an exaggerated increase in cerebral pulsatility among women and suggest a potential mechanism contributing to sex differences in brain aging.

## Graphical abstract

**Figure:**
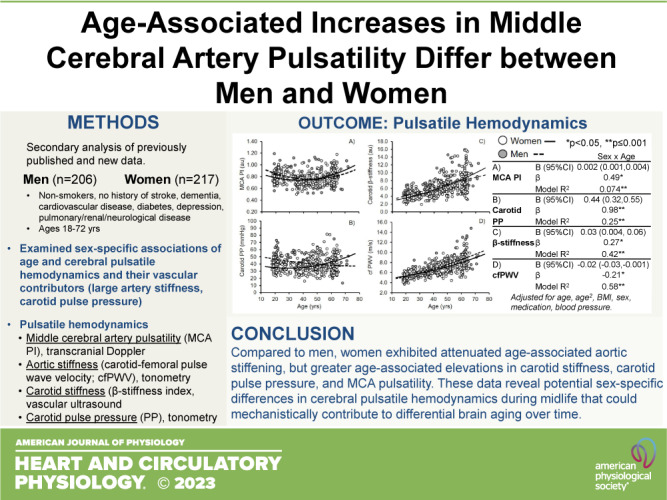

## INTRODUCTION

There are inherent sex differences in brain health with advancing age, with women bearing greater burden of cerebrovascular and cognitive disease (Alzheimer’s disease and related dementia) than men (1–4). The exact mechanisms underlying the sex differences in brain aging and cerebrovascular/cognitive disease risk are unknown but may be related to the pulsatile manner in which blood flow is delivered to the brain (5–8). Cerebral pulsatility describes the inherent temporal variation of blood flow within a cardiac cycle (e.g., systole vs. diastole) that increases with age in the sensitive cerebrovasculature owing to large artery (e.g., aorta and carotid) stiffening and expansion of pulse pressure (9). Higher cerebral pulsatility damages cerebral microvessels (10–13) and structures (11) and contributes to cerebrovascular disease and cognitive impairment (10–17). As such, cerebral pulsatility is an emerging target of brain health and potential mechanism contributing to sex differences in brain aging.

Recent data suggest that women may have greater cerebral pulsatility than men (9) and exhibit greater increases in cerebral pulsatility with aging (18). Although promising, early data were limited by sample size (19) and recent larger studies are limited by an imbalanced distribution of age [e.g., a bimodal distribution that largely excluded middle-aged adults (18), smaller number of younger adults (9), or linear analysis of pulsatility and age (18) despite prior evidence of a nonlinear relationship (9, 19)]. Understanding whether there are sex differences in cerebral pulsatility during early to midlife is critical as midlife in women coincides with menopause, a period of accelerated vascular aging (20–23). Moreover, vascular health in midlife in both sexes is predictive of later cerebrovascular and brain health (24–27). As such, the purpose of this study was to examine sex differences in age-associated changes in cerebral pulsatility, large artery stiffness, and carotid pulse pressure. To accomplish this, we leveraged our previously published cross-sectional cohort (9) and added newly collected data in 101 young and middle-aged adults to strengthen the cohort. We hypothesized that women would exhibit a greater age-associated change in cerebral pulsatility, aortic and carotid stiffness, and carotid pulse pressure compared with men.

## METHODS

This study is a secondary analysis that combines aspects of our prior datasets examining cerebrovascular hemodynamics in relation to age (9) and cardiorespiratory fitness (28) with new data from our laboratory. Cumulatively, these data (*n* = 423 in total) were collected between 2013 and 2023 across multiple institutions including Syracuse University (*n* = 239), University of Illinois at Chicago (*n* = 66), and Iowa State University (*n* = 118). All methods have been described in detail previously (9, 28) and are presented concisely here for the sake of brevity.

### Participants

Participants were generally healthy volunteers between the ages of 18 and 72 yr and were excluded based on self-reported smoking, stroke, dementia, diabetes mellitus, previous cardiovascular events, pulmonary/renal/neurological disease, depression, or head trauma (concussion) within previous 3 mo. Antihypertensive and statin medication use was self-reported. All participants provided written informed consent, and all study procedures were approved by the institutional review boards at their respective institutions and conformed to the standards outlined by the Declaration of Helsinki.

### Study Design

Cerebrovascular hemodynamics were assessed following ≥4 h fast and abstinence from caffeine/exercise the day of testing. Testing was standardized to the early follicular phase of the menstrual cycle or during the placebo week if taking oral contraceptives for 123 women, with testing not standardized in *n* = 8 menstruating women. Testing periods were not standardized for men, or women with >60 days of amenorrhea. All vascular measurements were obtained in a supine position in a dimly lit room after at least 10 min of quiet rest.

### Measures

#### Anthropometrics.

Body mass index (BMI) was derived using height and weight and measured using a stadiometer and electronic scale, respectively.

#### Brachial blood pressure.

Brachial systolic and diastolic blood pressure were measured via oscillometry or finger photoplethysmography calibrated to brachial blood pressure as described previously (28). Brachial mean arterial pressure was calculated as 1/3 systolic + 2/3 diastolic pressure.

#### Large artery stiffness.

Aortic stiffness was measured using carotid-femoral (cf) pulse wave velocity (cfPWV), the “gold standard” for central arterial stiffness measurements (29). Applanation tonometry (NIHem USB 2.0, Cardiovascular Engineering or SphygmoCor, AtCor Medical, Sydney, Australia) was used to assess carotid and femoral artery blood pressure waveforms over a 10- to 20-s epoch with electrocardiogram (ECG) for simultaneous R wave gating. cfPWV was calculated as the transit distance [suprasternal notch to carotid distance minus suprasternal notch to femoral distance (29)] between the pulse sites divided by the time delay between the cf waveforms. Carotid stiffness was measured below the carotid bulb using ultrasound (Aloka ProSound α7 and Arietta 70; Hitachi Healthcare Americas, Twinsburg, OH) with a 7.5- to 10.0-Hz linear-array probe. Five to seven distension waveforms were traced via onboard eTracking software and ensemble averaged for derivation of carotid β-stiffness as follows: ln(P_max_/P_min_)/[(*D*_max_ − *D*_min_)/*D*_min_], where P and *D* correspond to pressure and diameter, respectively, and maximum (max; systolic) and minimum (min; diastolic) values during the cardiac cycle.

#### Carotid pulse pressure.

Carotid artery pressure was collected as described earlier via applanation tonometry. Carotid pressure waves were captured over a 10- to 20-s epoch and ensemble averaged and calibrated based on best practices for each device: *1*) calibrated to brachial mean and diastolic pressure (SpygmoCor, ATCOR Medical, Sydney, Australia) or *2*) aligned with and calibrated to brachial pressure waveforms (NIHem USB 2.0, Cardiovascular Engineering). Carotid pulse pressure was calculated as systolic pressure minus diastolic pressure.

#### Middle cerebral artery pulsatility.

Middle cerebral artery (MCA) pulsatility index (PI) was measured via transcranial Doppler (DWL Doppler BoxX, Compumedics, Germany; TOCM Nuerovision, Multigon Industries, Elmsford, NY) and a 2-MHz probe secured to the left temporal window. MCA PI was collected over a 12- to 45-s epoch and calculated using peak systolic (*V*_s_), diastolic (*V*_d_), and mean velocity (*V*_mean_) as PI = (*V*_s_ − *V*_d_)/*V*_mean_.

### Statistical Analyses

All data are reported as means ± SD, with significance set as *P* < 0.05. Descriptive characteristics and cerebrovascular hemodynamics were compared between men and women using unpaired *t* tests. The effects of age and sex on cerebral pulsatility (MCA PI), aortic stiffness (cfPWV), carotid stiffness (β-stiffness) and pulse pressure, and MCA mean velocity (see Supplemental Table S1; https://doi.org/10.6084/m9.figshare.24000198) were tested via multiple regression and the enter method. *Model 1* included age, age^2^ [owing to nonlinear association between vascular outcomes and age established previously (9, 19, 30)], sex, and a sex-by-age interaction term used to identify sex-specific differential relationships between age and vascular outcomes. *Model 2* was further adjusted for body mass index, use of antihypertensive therapy, use of statin therapy, and mean arterial pressure. A sensitivity analysis was conducted for MCA PI (*model 3*) wherein carotid pulse pressure was further adjusted owing to its mechanistic contribution to cerebral pulsatility. All analyses were performed using Statistical Package for the Social Sciences (SPSS, version 25, IBM, Chicago, IL).

## RESULTS

### Subject Characteristics and Cerebrovascular Hemodynamics

Mean age, antihypertensive therapy use, statin therapy use, BMI, MCA pulsatility index, brachial mean and diastolic pressure, and aortic and carotid stiffness were not statistically different between men and women (Table 1). Women on average had a greater MCA mean velocity and lower brachial and carotid systolic pressures, and carotid pulse pressure compared with that of men (*P* < 0.05).

**Table 1.** Descriptive characteristics of the study sample

	Men		Women		
		*n*		*n*	Combined
Age, yr	43 ± 16	206	43 ± 15	217	43 ± 16
Anti-HTN therapy, *n* (%)	21 (10.2)	206	22 (10.1)	217	43 (10.1)
Statin therapy, *n* (%)	35 (17.0)	206	27 (12.4)	217	62 (14.7)
Oral contraceptive use, *n* (%)		206	27 (12.4)	217	
Height, m	1.77 ± 0.07	206	1.64 ± 0.07*	217	1.71 ± 0.09
Weight, kg	82.4 ± 13.2	206	70.2 ± 12.7*	217	76.2 ± 14.3
BMI, kg/m^2^	26.3 ± 3.7	206	26.0 ± 4.3	217	26.1 ± 4.0
MCA mean velocity, cm/s	60 ± 15	191	68 ± 17*	208	64 ± 17
MCA pulsatility index, AU	0.79 ± 0.13	190	0.78 ± 0.11	208	0.79 ± 0.12
Brachial artery, mmHg					
SBP	124 ± 10	204	119 ± 14*	216	121 ± 12
DBP	74 ± 9	204	74 ± 9	216	74 ± 9
MAP	92 ± 9	204	91 ± 10	216	91 ± 10
Carotid artery					
SBP, mmHg	116 ± 12	204	113 ± 15*	216	114 ± 14
PP, mmHg	41 ± 10	204	38 ± 12*	216	40 ± 11
β-Stiffness, AU	6.4 ± 2.4	204	6.2 ± 2.9	216	6.3 ± 2.7
cfPWV, m/s	7.1 ± 1.9	206	6.8 ± 1.6	217	6.9 ± 1.7

Values are means ± SD; *n* = 423 participants. HTN, hypertension; BMI, body mass index; MCA, middle cerebral artery; AU, arbitrary units; SBP, systolic blood pressure; DBP, diastolic blood pressure; MAP, mean arterial blood pressure; PP, pulse pressure; cfPWV, carotid-femoral pulse wave velocity. *P < 0.05 vs. men via unpaired *t* test.

### Age-Associated Changes in Cerebral Pulsatility, Large Artery Stiffness, and Carotid Pulse Pressure

**Figure 1.**
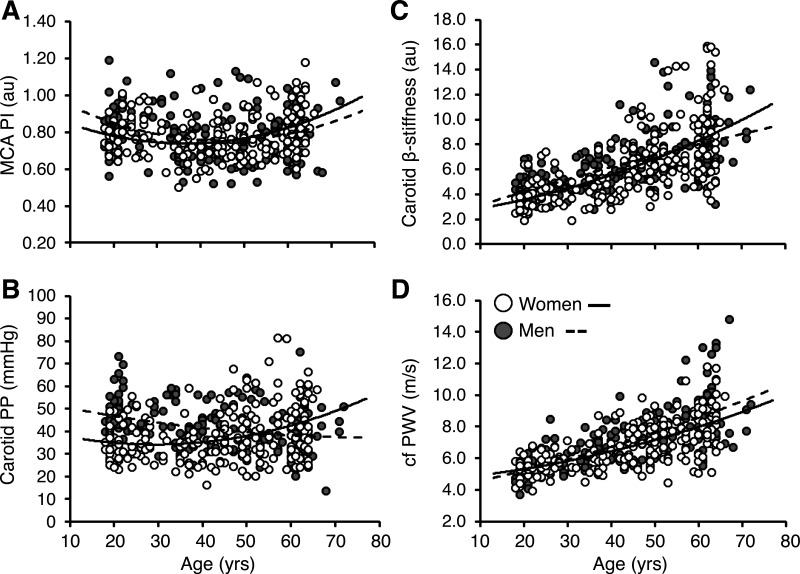
Relationship between age and middle cerebral artery (MCA) pulsatility (PI, pulsatility index; *n* = 189 men, *n* = 207 women; *A*), carotid pulse pressure (PP; *n* = 203 men, *n* = 216 women; *B*), and carotid β-stiffness (*n* = 201 men, *n* = 216 women; *C*), and aortic stiffness (cfPWV, carotid-femoral pulse wave velocity; *n* = 203 men, *n* = 214 women; *D*) for men and women. Data represent *model 1* from multiple regression analyses.

The sex-by-age interaction term significantly predicted MCA pulsatility after adjusting for age, age^2^, and female sex (*model 1*; adjusted *R*^2^ = 0.074, *P* < 0.001; Table 2 and Fig. 1*A*) and remained significant after further adjustment for antihypertensive therapy, statin therapy, BMI, and mean arterial pressure (*model 2*; adjusted *R*^2^ = 0.076, *P* < 0.001). The sex-by-age interaction term was no longer a significant predictor of MCA pulsatility after including carotid pulse pressure (*model 3*; adjusted *R*^2^ = 0.27, *P* < 0.001). Aortic stiffness, carotid stiffness, and carotid pulse pressure were significantly predicted by the sex-by-age interaction term in *model 1* (Fig. 1, *B*–*D*), and remained significant after further adjusting for antihypertensive therapy, statin therapy, BMI, and mean arterial pressure in *model 2* (Table 3; aortic stiffness, adjusted *R*^2^ = 0.58; carotid stiffness, adjusted *R*^2^ = 0.42; carotid pulse pressure, adjusted *R*^2^ = 0.25; *P* < 0.001 for all). *Model 2* results for MCA mean velocity were similar and are displayed in Supplemental Table S1.

**Table 2.** Multiple regression assessing interactions between age and sex on MCA pulsatility in 396 adults

	*Model 1*		*Model 2*		*Model 3*	
	B (95% CI)	β	B (95% CI)	β	B (95% CI)	β
Age	−0.01 (−0.02, −0.01)	−1.71**	−0.01 (−0.02, −0.01)	−1.63**	−0.01 (−0.01, −0.004)	−1.07**
Age^2^	1.50 (0.90, 2.10)	1.61**	1.50 (0.90, 2.10)	1.60**	1.20 (0.60, 1.70)	1.24**
Female sex	−0.12 (−0.19, −0.05)	−0.49**	−0.12 (−0.19, −0.05)	−0.48**	0.001 (−0.07, 0.07)	0.00
Sex × age	0.003 (0.001, 0.004)	0.51**	0.002 (0.001, 0.004)	0.49*	0.00 (−0.001, 0.002)	0.02
BMI			−0.001 (−0.004, 0.002)	−0.05	0.001 (−0.002, 0.003)	0.02
MAP			−0.001 (−0.002, 0.00)	−0.08	−0.004 (−0.005, −0.002)	−0.29**
CCA PP					0.006 (0.005, 0.007)	0.50**
Adjusted *R*^2^	0.074		0.074		0.264	
Adjusted Δ*R*^2^	0.074		0.00		0.19	
*F* statistic	8.88		5.00		16.76	

Males, *n* = 189; females, *n* = 207. Age^2^ unstandardized beta is reported as ×10^−4^. Antihypertensive therapy and statin therapy use included as covariates in *models 2* and *3* but are not reported. B, unstandardized beta weight; MCA, middle cerebral artery; CI, confidence interval; BMI, body mass index; MAP, mean arterial pressure; CCA PP, common carotid artery pulse pressure. Significant predictor at *P < 0.05 and **P ≤ 0.001.

**Table 3.** Multiple regression assessing interactions between age and sex on aortic stiffness, carotid pulse pressure, and carotid stiffness

	cf Pulse Wave Velocity^a^		CCA Pulse Pressure^b^		CCA β-Stiffness^c^	
	B (95% CI)	β	B (95% CI)	β	B (95% CI)	β
Age	0.01 (−0.04,0.06)	0.13	−0.85 (−1.26,−0.45)	−1.22**	0.06 (−0.03, 0.14)	0.32
Age^2^	0.001 (0.00,0.001)	0.48*	0.01 (0.002,0.01)	0.83*	0.00 (−0.001, 0.001)	0.13
Female sex	0.40 (−0.25,1.04)	0.11	−21.82 (−27.22,−16.42)	−1.00**	−1.48 (−2.64, −0.31)	−0.28*
Sex × age	−0.02 (−0.03,−0.001)	−0.21*	0.44 (0.32,0.55)	0.98**	0.03 (0.004, 0.06)	0.27*
BMI	0.03 (−0.003,0.06)	0.06	−0.32 (−0.55,−0.08)	−0.12*	−0.05 (−0.10, 0.001)	−0.08
MAP	0.05 (0.04,0.06)	0.28**	0.46 (0.36,0.57)	0.41**	0.05 (0.03, 0.07)	0.18**
Adjusted *R*^2^	0.58		0.25		0.42	
*F* statistic	72.13		18.42		39.16	

Participants were studied with aortic stiffness (*n* = 417), carotid pulse pressure (*n* = 419), and carotid stiffness (*n* = 418). ^a^Men, *n* = 203; women, *n* = 214. ^b^Men, *n* = 203; women, *n* = 216. ^c^Men, *n* = 202; women, *n* = 216. Antihypertensive therapy and statin therapy use included as covariates in model but are not reported. B, unstandardized beta weight; CCA, common carotid artery; CI, confidence interval; BMI, body mass index; MAP, mean arterial pressure. Significant predictor at *P < 0.05 and **P ≤ 0.001.

## DISCUSSION

This secondary analysis aimed to examine sex differences in age-associated changes in aortic and carotid stiffness, carotid pulse pressure, and cerebral pulsatility. The primary observations from this cross-sectional analysis were that compared with men *1*) women exhibited attenuated age-associated increases in aortic stiffness but exaggerated age-associated increases in carotid stiffness and *2*) women exhibited greater age-associated increases in cerebral pulsatility, driven in part by greater age-associated increases in carotid pulse pressure. These cross-sectional data suggest accelerated age-associated changes in pulsatile hemodynamics in women during midlife. Taken together, our data indicate that there are sex differences in age-associated increases in cerebral pulsatility, an emerging vascular target of brain health that could contribute to sex differences in brain health with aging.

We observed greater age-associated aortic stiffening in men versus women, which remained statistically significant after adjustment for confounding variables. Data examining sex differences in aortic stiffness (cfPWV) across the life span are somewhat mixed, with longitudinal data suggesting men exhibit greater increases in cfPWV with aging (31) or that women exhibit greater increases, but remain lower than men with aging (30). Cross-sectional data, however, have reported no differences in associations between age and cfPWV between men and women across the life span (32–34). Of note, data indicate that aortic stiffening (assessed as cfPWV) is associated with cognitive dysfunction in older men but not in older women (35), potentially suggesting that aortic stiffening (i.e., cfPWV) may not be as strongly linked to brain health in women. Although our cross-sectional data suggest men exhibited greater age-associated increases in aortic stiffness, women in our sample exhibited greater age-associated increases in carotid stiffness. Observations of sex differences (or lack thereof) within the large elastic artery stiffness and aging literature may depend, in part, on the segments of the arterial tree examined [e.g., brachial-ankle PWV (36) versus cfPWV versus proximal aortic stiffness (37)]. Regional measures of aortic (e.g., characteristic impedance and β-stiffness) (37) and carotid stiffness (38) appear to increase more drastically with age among women compared with men, potentially due to menopausal transition during midlife and changes in the sex hormone environment (20, 39). Ultimately, the proximity of the carotid artery to the sensitive cerebrovasculature and its apparent accelerated age-associated stiffening among women compared with men could contribute to differential cerebrovascular pulsatile hemodynamics among women.

Our cross-sectional data suggest that women exhibit greater age-associated increases in cerebral pulsatility at the level of the MCA compared with that of men. This observation aligns with recent cross-sectional data by Alwatban et al. (18) in a substantial sample of adults across the life span; however, their data were limited by a linear analysis and bimodal age distribution with little data spanning early midlife (e.g., 40–50 yr of age). Our earlier cross-sectional work noted that, on average, females had greater cerebral pulsatility than males (9), but we had a limited distribution of younger adults. Earlier studies noting no sex differences in cerebral pulsatility were limited by sample size (19). Our analyses suggest that greater age-associated changes in cerebral pulsatility in women may be related to exaggerated age-associated increases in carotid pulse pressure compared with men. Greater expansion of pulse pressure with aging in women compared with men, especially during midlife, has been shown previously in robust datasets (30, 34, 40, 41). Both longitudinal and cross-sectional data from the Framingham Heart Study suggest that the greater age-associated increase in pulse pressure among women stems from exaggerated increases in aortic characteristic impedance and forward wave amplitude compared with men (30, 42, 43). Our data connect these observations to sex differences in cerebral hemodynamics with aging whereby greater increases in large artery characteristic impedance with aging may amplify forward wave transmission among women, thus increasing carotid pulse pressure and cerebral blood velocity pulsatility compared with men. This sex-specific augmentation of cerebral pulsatile transmission with aging may be exaggerated by inherent sex differences in the ability of the cerebrovasculature to damp (i.e., attenuate) pulsatile blood flow (9). Ultimately, greater pulsatile blood velocity and pressure in the cerebrovasculature have been linked to cerebrovascular and structural damage in the brain (7, 10, 14) and may contribute to accelerated brain aging and disproportionate cerebrovascular and cognitive disease risk among women.

### Implications and Future Directions

Our cross-sectional data suggest that cerebrovascular pulsatile hemodynamics and their mechanistic contributors increase rapidly with age during early midlife (40–50 yr) in women, which aligns with previous observations (30, 39) and coincides with the menopause transition (44). The menopause transition is accompanied by fluctuations and ultimately declines in estrogen and increases in follicle-stimulating hormone, which have been associated with greater proximal aortic stiffness in women (39), and the menopause transition may accelerate carotid stiffening and expansion of pulse pressure in women (20). This may be of particular importance since greater pulsatile pressure has been linked with greater damage to white matter and subcortical microstructures within the brain in women (35). Our data suggest that exaggerated increases in cerebral pulsatility, driven by the rapid expansion of pulse pressure in the cerebrovasculature among women, may contribute to accelerated brain aging and disproportionate cerebrovascular and cognitive disease risk in this group. As such, mechanistic changes in large artery characteristic impedance, carotid stiffness, and ultimately pulsatile cerebral hemodynamics during the menopause transition may serve as a key underlying mechanism contributing to sex differences in brain health with advancing age. Currently, this area is woefully under investigated (45) despite its emerging importance for understanding, and ideally attenuating, sex differences in cerebrovascular and brain aging. We additionally wish to underscore that the current sample included generally healthy adults and adults with cardiovascular disease risk factors (e.g., hypertension, obesity, and dyslipidemia), but excluded adults with chronic diseases such as stroke, dementia, diabetes mellitus, and neurological disease. As such, our observation of differential age-related changes in pulsatile hemodynamics between men and women may only be present in our specific sample and may differ in other populations. Future work should examine sex-specific relations between age and cerebral pulsatile hemodynamics in populations with greater risk factor burden or chronic diseases to better understand the influence of these conditions on sex-specific cerebrovascular aging trajectories.

### Limitations

These data are cross-sectional in nature and although suggestive of rapid changes in cerebral pulsatility in women during midlife, a period of aging that coincides with the menopausal transition, longitudinal data are necessary to adequately characterize changes in pulsatility during this aging window. This dataset is also limited by a small number of perimenopausal individuals (*n* = 14, ∼6.4% of women in the sample), who have often been excluded from previous study samples examining cerebrovascular hemodynamics (45). Because of the secondary nature of this analysis and limited perimenopausal sample, we are not adequately positioned to fully delineate the role of the menopause transition stage on cerebrovascular hemodynamics. In addition, the vast majority of datasets compiled as part of this secondary analysis unfortunately did not include serum sex hormones or detailed characterization of menopausal status, limiting our ability to examine the role of age-associated changes in gonadal status within this sample. Future work examining cerebrovascular pulsatile hemodynamics during midlife specifically should include characterization of sex hormones including follicle-stimulating hormone, luteinizing hormone, progesterone, testosterone, estrone, and estradiol, owing to their potential influence on cerebrovascular hemodynamics. We wish to underscore that beta-weights from cross-sectional analyses provide estimates of rates of change in a particular outcome for a given change in age, but are not truly longitudinal data that provide insight into within-person rates of change over time. Finally, the age range was slightly larger in the sample of men compared with women (maximum age of 72 vs. 65 yr, see Fig. 1), which has the potential to exaggerate or attenuate the sex-specific relations between age and vascular outcomes herein. We do not believe that the slight differences in age distribution between men and women substantially altered our findings since sex-by-age interaction terms for all vascular outcomes remained statistically significant after excluding males >65 yr of age as part of an exploratory analysis (data not shown).

### Conclusions

Our data suggest that compared with men, women exhibit attenuated age-associated aortic stiffening but exaggerated age-associated elevations in carotid stiffness, carotid pulse pressure, and MCA pulsatility. Moreover, our data suggest that greater age-associated elevations in MCA pulsatility in women may stem from the exaggerated expansion of pulse pressure within this group, particularly during midlife. Cumulatively, these data reveal the potential sex-specific alterations in cerebral pulsatile hemodynamics during midlife that could mechanistically contribute to differential brain aging in men versus women over time; however, additional longitudinal work is necessary to test this hypothesis.

## DATA AVAILABILITY

The raw data supporting the conclusions of this manuscript are available from the corresponding author upon reasonable request.

## SUPPLEMENTAL DATA

10.6084/m9.figshare.24000198 Supplemental Table S1: https://doi.org/10.6084/m9.figshare.24000198.

## GRANTS

The analyses presented in this manuscript were not specifically funded. The data compiled within this secondary analysis were funded in part by an American College of Sports Medicine foundation research grant, American Heart Association Predoctoral Fellowships 6PRE31220031 and 19PRE34380420, Syracuse University Sydney Young Research Award, Syracuse University School of Education Creative Grant Award, National Institutes of Health (NIH) Grants T32HL134634 and R03MD-011306-02, and Iowa State University College of Human Sciences faculty seed grant.

E. Lefferts’ contributing studies were supported in part by American Heart Association Predoctoral Fellowship 19PRE34380420. K. Heffernan’s contributing studies were supported in part by the Dairy Research Institute/Dairy Management, Inc., Grant 1154 and NIH Grant R03 MD-011306-02.

## DISCLAIMERS

The content is solely the responsibility of the authors and does not necessarily represent the official views of the National Institutes of Health.

## DISCLOSURES

No conflicts of interest, financial or otherwise, are declared by the authors.

## AUTHOR CONTRIBUTIONS

W.K.L. conceived and designed research; W.K.L. and J.A.A. performed experiments; W.K.L., K.S.R., and R.E.R. analyzed data; W.K.L., K.S.R., R.E.R., and K.L.M. interpreted results of experiments; W.K.L., K.S.R., and R.E.R. prepared figures; W.K.L., K.S.R., and R.E.R. drafted manuscript; W.K.L., K.S.R., R.E.R., J.A.A., and K.L.M. edited and revised manuscript; W.K.L., K.S.R., R.E.R., J.A.A., and K.L.M. approved final version of manuscript.
